# Usefulness of apparent diffusion coefficient values and magnetic resonance imaging histogram analysis for identifying histological types of preoperative testicular tumors

**DOI:** 10.1186/s12894-025-01825-4

**Published:** 2025-05-28

**Authors:** Yuka Yasuda, Akiyoshi Osaka, Keita Izumi, Toshiyuki Iwahata, Akinori Nakayama, Kazunori Kubota, Kazutaka Saito

**Affiliations:** 1 Department of Urology, Dokkyo Medical University Saitama Medical Center, Koshigaya, Japan; 2 Department of Radiology, Dokkyo Medical University Saitama Medical Center, Koshigaya, Japan

**Keywords:** Preoperative testicular tumor, Magnetic resonance imaging, Seminoma, Malignant lymphoma, Apparent diffusion coefficient

## Abstract

**Background:**

Only a few studies have performed histogram analysis for the differential diagnosis of testicular tumors. Therefore, the aim of this study was to evaluate the ability of magnetic resonance imaging, including diffusion-weighted imaging with apparent diffusion coefficient values, to differentiate between the histological types of testicular tumors.

**Methods:**

Of the 156 testicular tumors diagnosed at our hospital between January 2010 and July 2023, 65 cases diagnosed with magnetic resonance imaging were included. Tumors were categorized as seminoma, non-seminoma, and malignant lymphoma. Apparent diffusion coefficient values were calculated and analyzed using the ratio to non-tumor testes and histograms according to tumor subtypes.

**Results:**

Among the 65 cases, 46, 14, and 5 entailed seminomas, non-seminomas, and malignant lymphomas, respectively. The apparent diffusion coefficient value ratio of seminomas (0.745 ± 0.132) was significantly higher than that of malignant lymphomas (0.531 ± 0.119, *p* = 0.013), and the apparent diffusion coefficient value ratios of non-seminomas (1.197 ± 0.430) were significantly higher than those of seminomas and malignant lymphomas (*p* = 0.0013 and *p* < 0.001, respectively). Seminomas and malignant lymphomas had significantly higher kurtosis values (8.55 ± 5.76 and 18.11 ± 5.22, respectively) than non-seminomas (4.92 ± 3.85, *p* = 0.012 and *p* = 0.0022, respectively). Malignant lymphomas had significantly higher kurtosis values than seminomas (*p* = 0.0123). Seminomas and malignant lymphomas had significantly higher skewness values (1.77 ± 1.00 and 3.12 ± 0.28, respectively) than non-seminomas (0.52 ± 1.17, *p* = 0.0016 and *p* < 0.001, respectively). Malignant lymphomas had higher skewness than seminomas (*p* < 0.001).

**Conclusions:**

The present results demonstrate the efficacy of magnetic resonance imaging with apparent diffusion coefficient values and histograms in the differentiation of testicular tumor subtypes. A pre-operative diagnosis of testicular tumor subtypes may enable more effective management of testicular tumors, including pre-operative counseling and early treatment planning.

## Background

Testicular tumors are heterogeneous and include various histological subtypes, including seminoma and non-seminoma. Malignant lymphoma occurs in the testis without lymph node involvement [1]. Although histological subtypes are determined through pathological evaluation, preoperative evaluation is crucial for developing appropriate treatment plans [2]. Imaging-based testicular tumor diagnosis for tumor presence and qualitative evaluation, including the tumor histological subtype, are essential. Magnetic resonance imaging (MRI) enables detailed internal anatomical and qualitative assessments and is useful in diagnostic imaging, which is widely used in clinical settings [3–7]. However, detailed studies using MRI for the differential diagnosis of the histological subtypes of testicular tumors are lacking. Diffusion-weighted imaging (DWI) in MRI produces an apparent diffusion coefficient (ADC) map via ADC values and is useful in cancer diagnosis [8–11]. Tumor tissues demonstrate low ADC values owing to the restriction of free extracellular water molecule diffusion caused by increased cancer cell density and irregular growth [8]. Recently, histogram analysis has attracted considerable attention. However, only a few studies have performed histogram analysis for the differential diagnosis of testicular tumors. Therefore, we aimed to investigate the usefulness of MRI for testicular tumors using ADC values and histogram analysis for each histological subtype of testicular tumor.

## Methods

### Patients

This retrospective study was approved by the Bioethics Committee of Dokkyo Medical University Saitama Medical Center (approval number: 24007). An information release regarding this study has been published on the university’s website. No patients involved in this study requested to withdraw their participation. The Bioethics Committee waived the requirement for informed consent owing to the retrospective nature of the study. Specifically, the planned study period spans from March 21, 2024, to March 31, 2025, and data were accessed between March 21, 2024, and October 26, 2024. During data collection, the authors had access to information that could identify individual participants. Of the 156 consecutive testicular tumors diagnosed at our hospital between January 2010 and July 2023, 77 cases included an MRI with DWI performed before testicular resection. Among these, 69 were diagnosed as malignant testicular tumors, of which four cases involving bilateral testicular tumors or a single testis after orchiectomy were excluded to permit comparison between tumor-bearing and contralateral non-tumor testes in the same patients. Imaging findings revealed no cases of bilateral testicular tumors of malignant lymphoma included in the study. Cases with MRI without DWI were excluded from the study. The remaining 65 cases were included and classified as seminomas (*n* = 46), non-seminomas (*n* = 14), and malignant lymphomas (*n* = 5).

### MRI procedures and measuring ADC values

The MAGNETOM Avanto Dot Upgr 1.5T (SIEMENS, Munich, Germany), MAGNETOM Skyra 3.0T (SIEMENS), and Ingenia CX 3.0T (PHILIPS, Amsterdam, the Netherlands) MRI systems were used in this study. DWI was performed using echo-planar imaging with a b-value of 0 s/mm^2^ for Ingenia CX and 1,000 s/mm^2^ for MAGNETOM Avanto Dot Upgr and MAGNETOM Skyra. The b-values for each tumor are presented in Table 1.

As three different ADC measurement procedures were used in this study, the ADC values of non-tumor testes differed among them. Considering that the use of different MRI scanners and various b values may affect the consistency of ADC measurements, the ADC value ratio of tumor-bearing testes to non-tumor testes was calculated to account for this difference for each tumor. Non-tumor testes were defined as testes without abnormal findings on MRI imaging. The mean ADC values of tumor-bearing and non-tumor testes were measured by setting a region of interest (ROI) on the largest cross-section of the testis on the ADC map, regardless of the presence of hemorrhage or necrotic lesions, as distinguishing them from tumor tissue on MRI images is difficult. Further, a histogram analysis was performed to measure kurtosis and skewness.

**Table 1:** B-values for each tumor

b-value (s/mm^2^)	Seminoma	Non-seminoma	Malignant lymphoma
0	2	1	1
1000	44	13	4

### Statistical analysis

Data are presented as the mean ± standard deviation. Significant differences were determined using the Wilcoxon test. Significance was set at 0.05 and the Bonferroni correction was applied. The score model was performed with the age at diagnosis and ADC values to distinguish testicular malignant lymphomas from seminomas. The typical onset ages of malignant lymphoma are higher, typically in the 50s and 60s, than those of seminomas, which commonly occur in individuals in their 30s and 40s [12, 13]. The total scores for seminomas and malignant lymphomas were calculated by adding their ADC value ratios. A receiver operating characteristic analysis was performed with a cut-off value of 0.637 for the ADC value ratio, with a specificity and sensitivity of 0.800 and 0.804, respectively. A value of 1 was assigned for ages < 30 and > 50 years and 0 for ages 30–50 years. Similarly, a value of 1 was assigned for the ADC value ratio of > 0.637 and 0 for ≥ 0.637.

## Results

### Patient background characteristics

Patients’ age ranges were 24–59 (median, 38.7), 19–51 (median, 33.6), and 24–74 (median, 56.8) years for seminomas, non-seminomas, and malignant lymphomas, respectively. Table 2 presents the histological types of testicular tumors for each case. Of the 14 patients with non-seminoma, three had pure histology of embryonal carcinoma, germ cell tumor, or teratoma. The histology of the remaining 11 patients was mixed non-seminomas. All five patients with testicular malignant lymphoma had diffuse large B-cell lymphoma histology. Testicular volume was measured using the formula length x width x height x 0.71 [14] (Table 2).

**Table 2:** Patients’ background characteristics

	Seminoma	Non-seminoma	Malignant lymphoma
Number of cases	46	14	5
Age (years)	24–59(38.7)	19–51(33.6)	24–74(56.8)
T1WI	↓	↓	↓
T2WI	↓	↑+↓	↓
DWI	↑	↑+↓	↑
ADC	↓	↑+↓	↓
ADC values ratio	0.745 ± 0.132	1.197 ± 0.430	0.531 ± 0.119
95% confidence interval	0.705–0.784	0.948–1.445	0.383–0.679
Tumor testis volume (cm^3^)	53.46 ± 57.34	98.47 ± 133.47	87.96 ± 96.23
Non-tumor testis volume (cm^3^)	13.91 ± 6.10	12.90 ± 5.40	12.38 ± 1.25
LDH (U/L)	486.1 ± 1534.3	377.6 ± 298.5	356 ± 299.2
AFP (ng/mL)	2.44 ± 1.65	393.01 ± 1129.38	2.38 ± 1.60
β-hCG (mIU/mL)	2.07 ± 8.18	16.05 ± 55.59	0
intact hCG (mIU/mL)	6.85 ± 27.00	50.89 ± 83.29	0.475 ± 0.95

Data are presented as ranges (mean). High signal: ↑, low signal: ↓, T1WI: T1-weighted imaging, T2WI: T2-weighted imaging, ADC: apparent diffusion coefficient.

### Testicular MRI findings

**Fig. 1:**
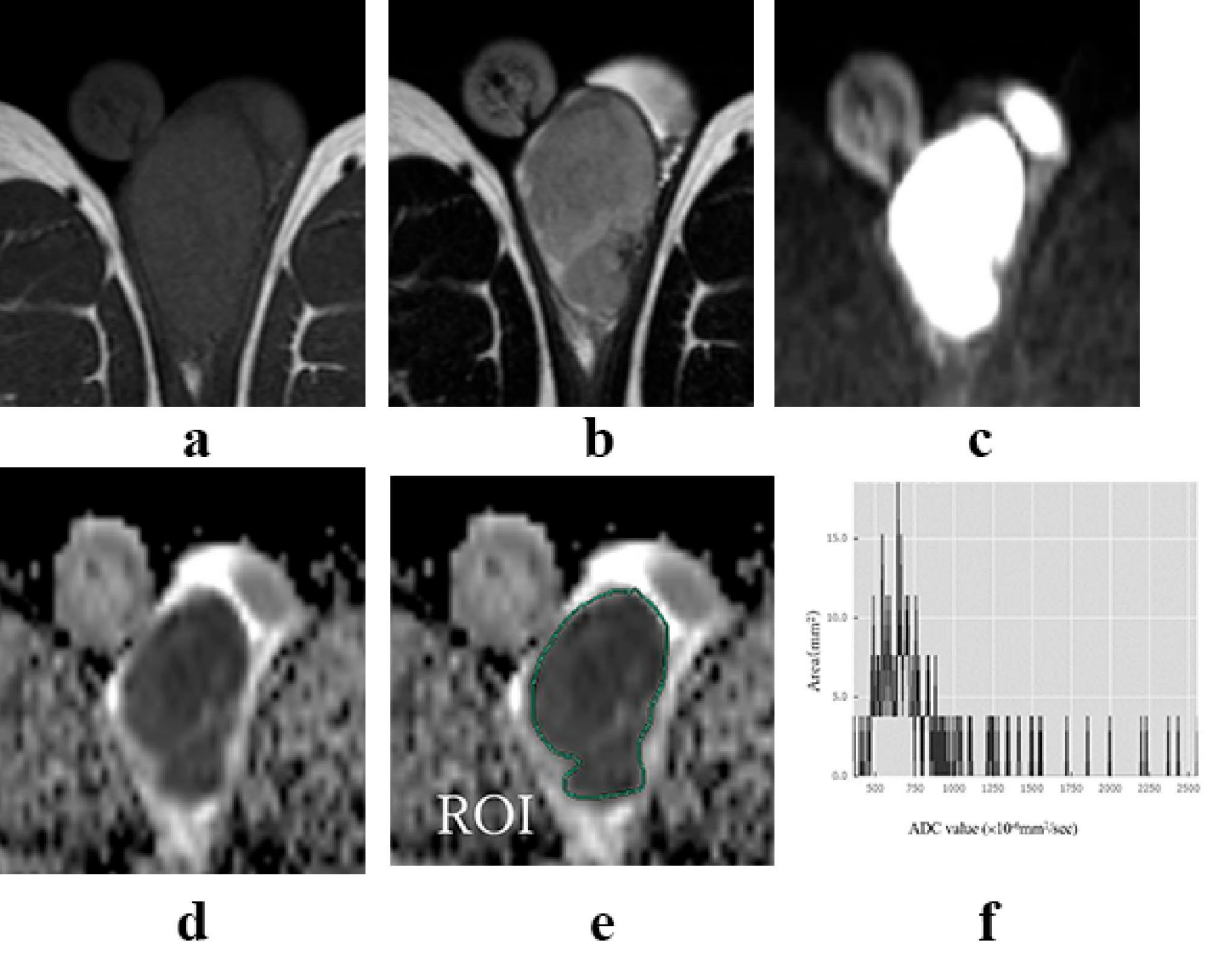
Representative findings of seminoma. T1W1 (**a**), T2W1 (**b**), DWI (**c**), and ADC map (**d**). The ROI surrounded the entire tumor on the ADC map (**e**). Histogram of ADC value (**f**). The histogram of the seminoma showed high kurtosis and skewness values of 15.318 and 3.21, respectively. It showed the distribution with peaked and heavy fringes compared with the normal distribution, and the mean ADC values were skewed toward the lower end of the distribution. T1WI: T1-weighted imaging, T2WI: T2-weighted imaging, ADC: apparent diffusion coefficient, ROI: region of interest, DWI: diffusion-weighted imaging

**Fig. 2:**
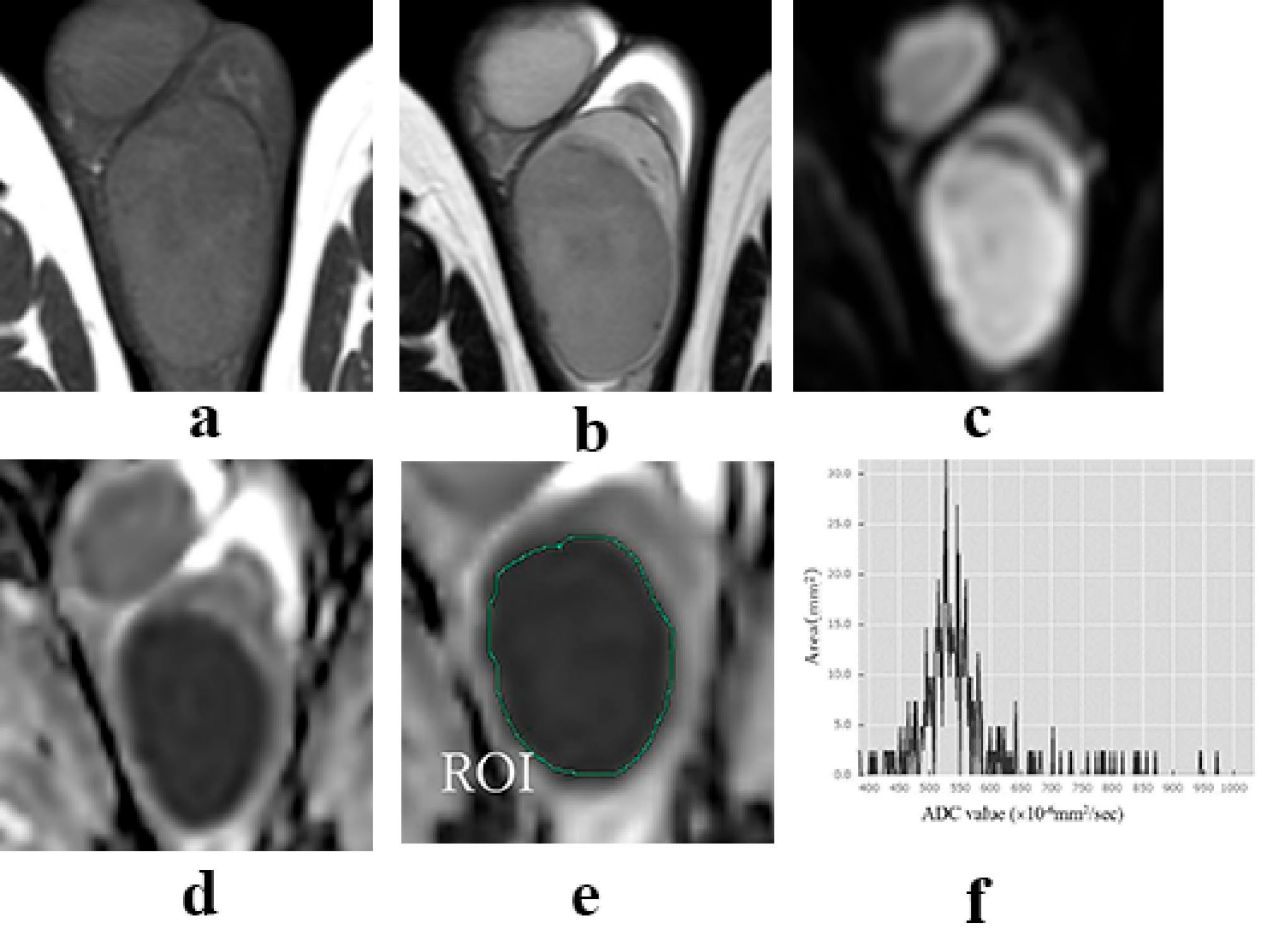
Representative findings of malignant lymphoma. T1W1(**a**), T2W1(**b**), DWI (**c**), and ADC map (**d**). The ROI surrounded the entire tumor on the ADC map (**e**). Histogram of the ADC value (**f**). The histogram of the seminoma showed high kurtosis and skewness values of 15.520 and 2.929, respectively. It had a shape with peaked peaks and heavy fringes compared with the normal distribution. The overall distribution of ADC values was skewed toward lower values than that of seminoma. T1WI: T1-weighted imaging, T2WI: T2-weighted imaging, ADC: apparent diffusion coefficient, ROI: region of interest, DWI: diffusion-weighted imaging

**Fig. 3:**
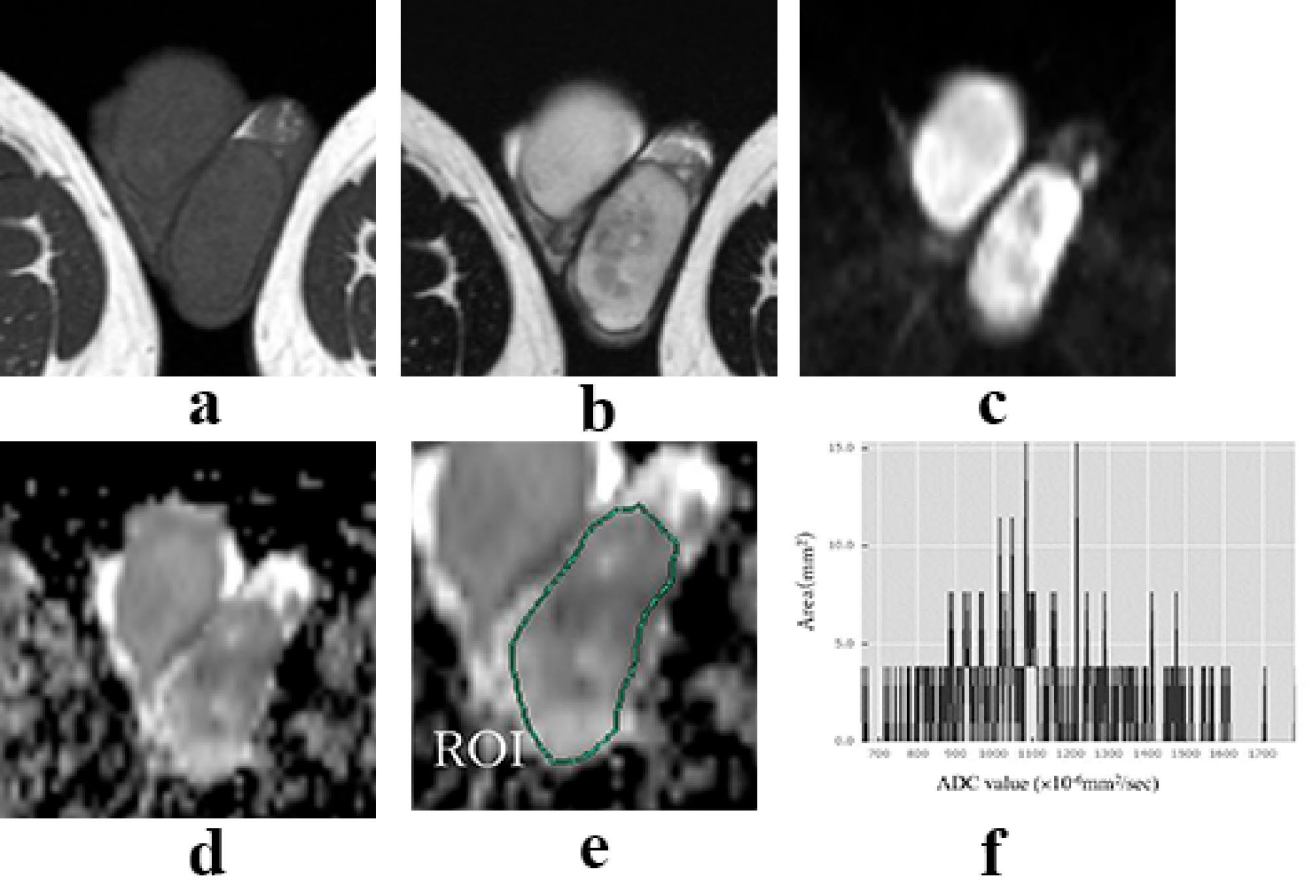
Representative findings of non-seminoma. T1W1(**a**), T2W1(**b**), DWI (**c**), and ADC map (**d**). The ROI surrounded the entire tumor region on the ADC map (**e**). Histogram of the ADC value (**f**). The case under consideration is that of a 27-year-old male patient. Preoperative tumor markers included LDH of 204 U/L, AFP of 2.9 ng/mL, β-HCG of 1.6 ng/mL, and intact HCG of 1.2. The histological types were identified as embryonal carcinoma, germ cell tumor, and seminoma. The histogram of the non-seminoma showed low kurtosis and skewness values of 2.632 and 0.464, respectively. It showed a heavy hem, the distribution was symmetrically close, and the mean ADC values showed a broad distribution. T1WI: T1-weighted imaging, T2WI: T2-weighted imaging, ADC: apparent diffusion coefficient, ROI: region of interest, DWI: diffusion-weighted imaging

Non-tumor testes showed low signal intensity on T1-weighted imaging (T1WI) and had high ADC values on MRI. Seminomas and malignant lymphomas showed low signal intensity on T2-weighted imaging (T2WI) and DWI (Figs. 1 and 2). Non-seminomas typically contained multiple tissues and did not show a single signal intensity (Fig. 3). However, seminomas and malignant lymphomas had similar signal intensities, making it difficult to distinguish them using T2WI or DWI (Figs. 1 and 2).

## ADC value ratio

**Fig. 4:**
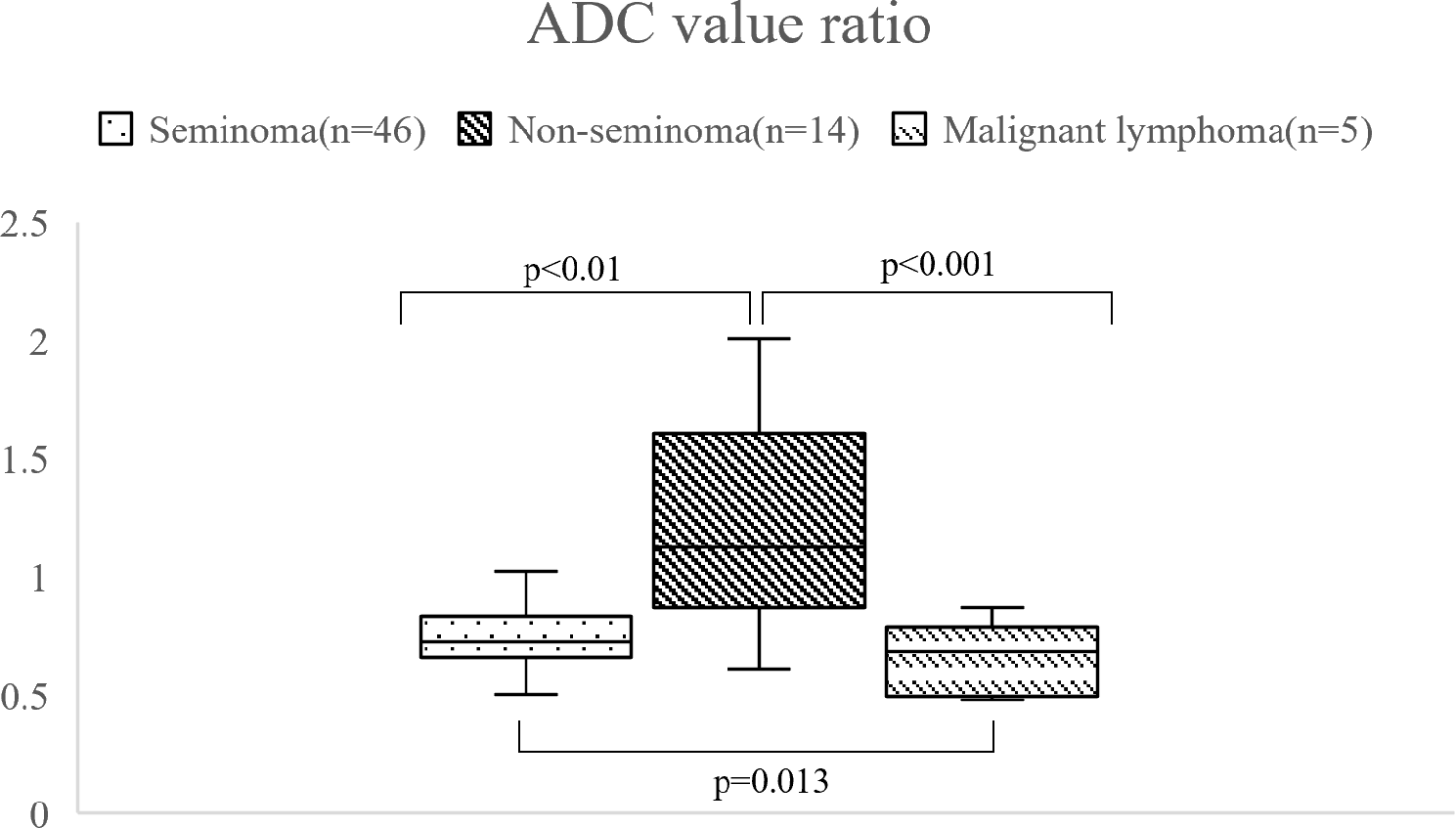
Distributions of the ADC value ratio. ADC: apparent diffusion coefficient

The ADC value ratio was 0.745 ± 0.132, 1.197 ± 0.430, and 0.531 ± 0.119 for seminomas, non-seminomas, and malignant lymphomas, respectively (Table 2). The ADC value ratio of seminomas was significantly higher than that of malignant lymphomas (*p* = 0.013), and the ADC value ratios of non-seminomas were significantly higher than those of seminomas and malignant lymphomas (*p* = 0.0013 and *p* < 0.001, respectively) (Fig. 4).

## The ADC histogram

**Fig. 5:**
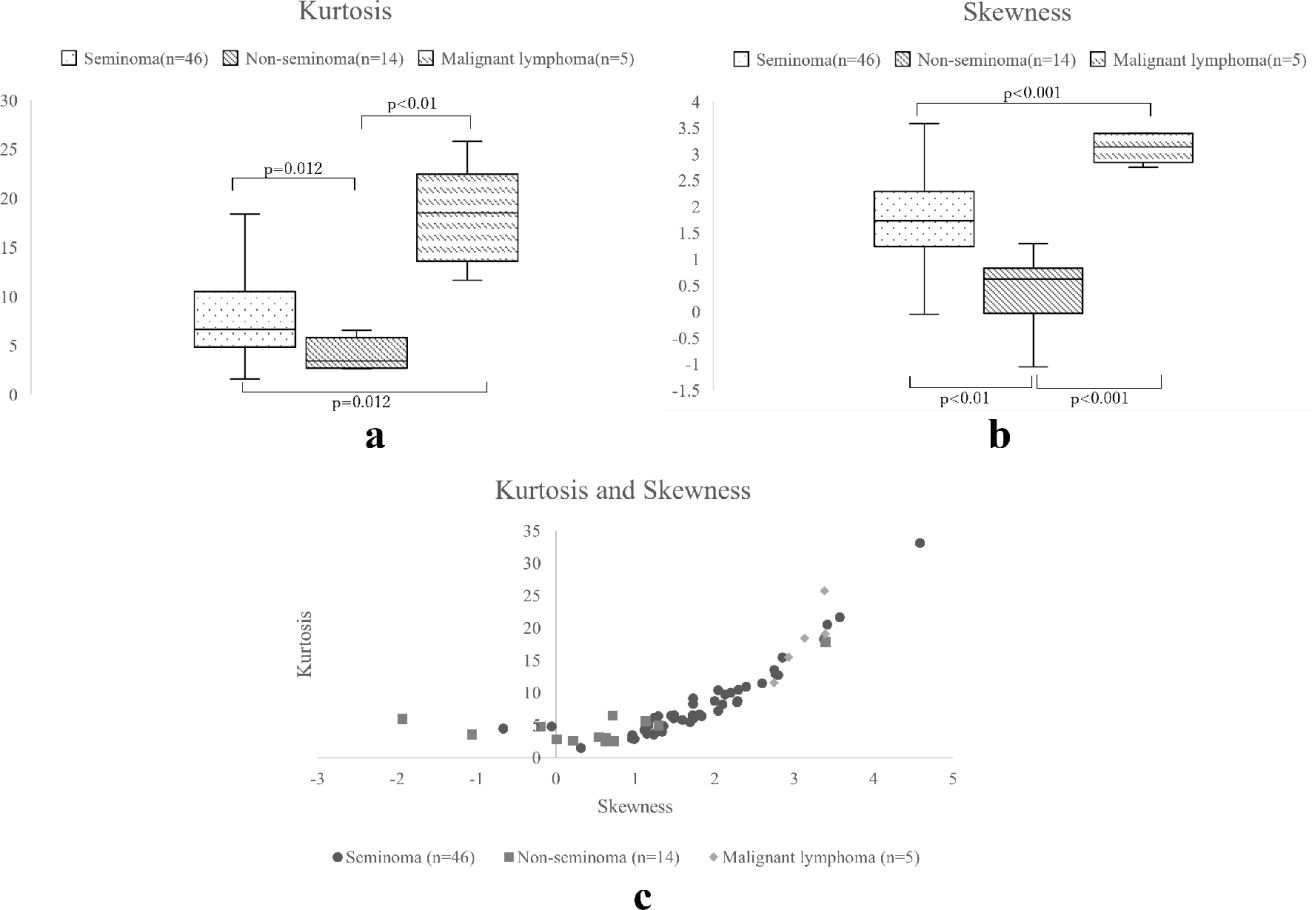
Kurtosis (**a**) and Skewness (**b**) of the ADC value histogram and correlation graph of skewness and kurtosis of the ADC value histogram (**c**). ADC: apparent diffusion coefficient

Kurtosis and skewness were measured from the ADC histograms. Kurtosis indicates the sharpness or heaviness of a data distribution base, and its value is high when the data are concentrated around the mean. Skewness denotes the degree of distribution asymmetry, and its value is high when the ADC value is skewed toward the lower side [8]. The kurtosis and skewness of tumor-bearing testes were compared, although the shooting conditions were different. Seminomas and malignant lymphomas had significantly higher kurtosis values (8.55 ± 5.76 and 18.11 ± 5.22, respectively) than non-seminomas (4.92 ± 3.85, *p* = 0.012 and *p* = 0.0022, respectively). Malignant lymphomas had significantly higher kurtosis values than seminomas (*p* = 0.0123). Seminomas and malignant lymphomas had significantly higher skewness values (1.77 ± 1.00 and 3.12 ± 0.28, respectively) than non-seminomas (0.52 ± 1.17, *p* = 0.0016 and *p* < 0.001, respectively). Malignant lymphomas had higher skewness than seminomas (*p* < 0.001) (Fig. 5).

### Scoring of seminomas and malignant lymphomas

**Fig. 6:**
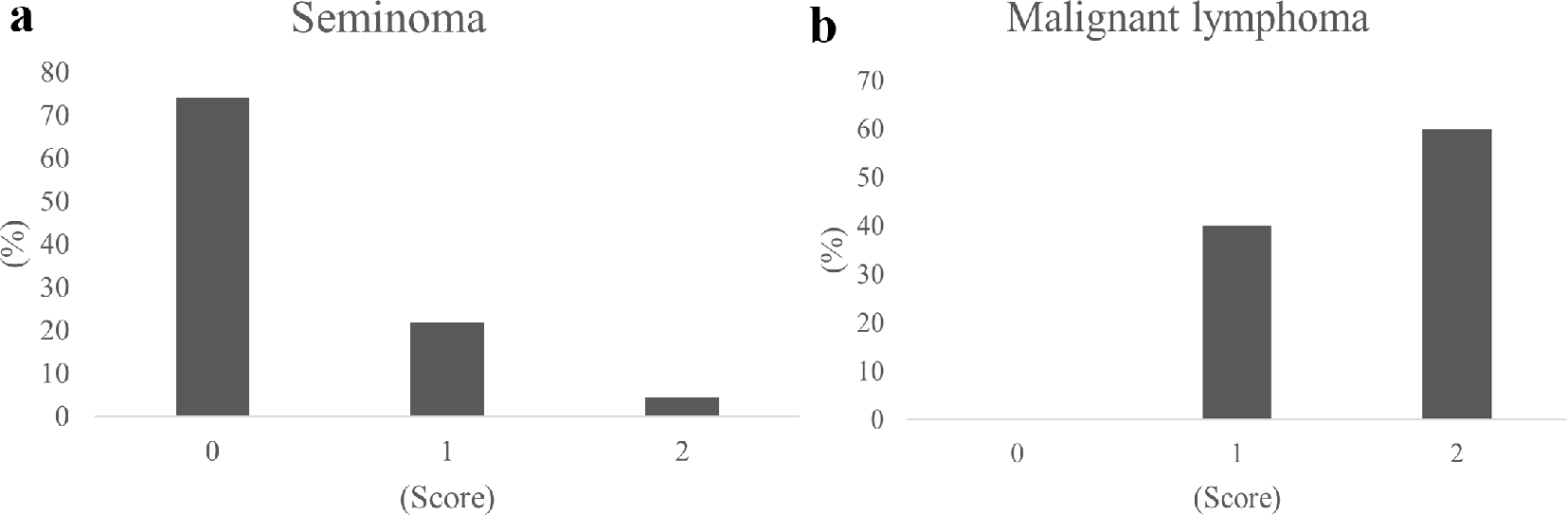
Probability of seminoma (**a**) and malignant lymphoma (**b**) according to the score

Using the scoring model with ADC value ratio and age, 34 (73.9%), 10 (21.7%), and 2 (4.3%) patients with seminomas scored 0, 1, and 2 points, respectively (Fig. 6a). In contrast, 0 (0%), 2 (40%) and 3 (60%) patients with malignant lymphomas scored 0, 1, and 2 points, respectively (Fig. 6b). In this scoring model, patients with scores of 0 and 1 and those with a score of ≥ 2 had a high probability of seminoma and malignant lymphoma, respectively.

## Discussion

This study indicated that MRI with ADC values and histograms are useful for characterizing testicular tumor subtypes. To our knowledge, this is the first study to demonstrate the significance of ADC values with histogram analysis in the histological types of testicular tumors. MRI provides detailed internal anatomical and qualitative evaluations. Moreover, DWI presents biological tissue properties using the water molecules’ diffusion phenomenon and ADC values as parameters to quantitatively evaluate the diffusion limit in vivo [8–11]. It reflects tumor cell density, and its usefulness has been reported at various tumor sites [12, 13]. We investigated the significance of ADC values in the histological types of testicular tumors using the ADC value ratio of the tumor-bearing testis to non-tumor testis as three different procedures were used in ADC measurement during the study period. Our study results demonstrate that seminomas and malignant lymphomas had lower ADC value ratio than non-seminomas, and malignant lymphomas had even lower ADC value ratios than seminomas. Non-seminomas display a wide distribution of ADC value ratios because they typically contain various histological types, and the ADC value ratios of each tissue type differ, as expected. Histogram analysis has recently attracted considerable attention. Moreover, ADC enables detailed tumor heterogeneity and cell density evaluation and aids histological diagnosis [15–20]. Kurtosis and skewness, statistical indices representing the distribution characteristics, were high in seminomas and malignant lymphomas in the ADC histogram analysis. Kurtosis of seminomas and malignant lymphomas were high, possibly reflecting cellular uniformity, compared with non-seminomas. Moreover, seminomas and malignant lymphomas showed low ADC values, which may have resulted in high skewness. Kurtosis and skewness were low in non-seminomas. Non-seminomas usually contain various histological types, and their ADC values were widely and moderately distributed, suggesting that they tend to have lower kurtosis and skewness than other malignant tumors. Testicular tumors include various subtypes categorized into seminomas or non-seminomas. Malignant lymphomas are also found as testicular tumors [21]. Although the diagnosis is made pathologically after the testis is resected, preoperative evaluation could be essential and beneficial in developing the treatment plan earlier [2]. Non-seminomas typically contain multiple histological types, including embryonal carcinomas, yolk sac tumors, choriocarcinomas, and teratomas, and distinguishing non-seminomas from seminomas or malignant lymphomas with a mixture of high- and low-signal areas on testicular MRI images can be challenging [22], and it makes difficult to strictly map each histology type of non-seminomas to MRI findings. The same applies to necrotic tissue, and because ROIs were extracted en bloc in the present study, the influence of necrotic tissue on ADC values cannot be ruled out. However, in this study, we aimed to distinguish the ADC value characteristics among seminoma, non-seminoma, and malignant lymphoma. Therefore, we selected the ROI on the largest tumor cross section including fibrotic or necrotic lesions although we recognized that this ROI setting could affect the results. These are considered to be issues for future research. On the histograms, kurtosis and skewness of non-seminomas were lower than those of seminomas and malignant lymphomas, as expected. Although it is beyond the scope of this study, a detailed analysis of each histological type would be warranted in the future. Preoperative differential diagnosis between seminoma and testicular malignant lymphoma is typically complex. The current results demonstrate their distinct characteristics based on DWI findings. As the ADC value ratios of seminomas and malignant lymphomas are lower than those of non-seminomas, we attempted to distinguish them by scoring them using age and ADC value ratio. Among patients with seminoma, 73.9% had the lowest score (0 points) for the possibility of seminoma. Of the malignant lymphoma cases, 60% had the highest score (2 points) for the possibility of malignant lymphoma. As all types included a case with a score of 1, studying a larger sample size, especially for malignant lymphoma, and reexamining the ADC ratio value, including its setting value, is considered necessary. We confirmed significant differences among histological types by measuring the ADC value ratio, analyzing ADC histograms, and scoring seminomas and malignant lymphomas based on age and ADC value ratio. These values can be used as an indicator for MRI diagnosis in cases such as ours, where determining the histological type is challenging. Furthermore, the detailed understanding of imaging findings may further elucidate testicular tumor biology. Currently, testicular MRI does not have a clear diagnostic superiority over ultrasonography and has not been positioned as a standard imaging modality. However, in the future, with further research and technological advancements, the role of MRI in the diagnosis of testicular tumors may expand. Therefore, with the accumulation of more research and clinical experience, the usefulness of testicular MRI is expected to become clearer, contributing to improved diagnostic accuracy and decision-making in treatment strategies.

This study had some limitations that must be acknowledged. First, the small sample and the rarity of testicular tumors, which occur in only 3–10 per 100,000 males [23], limited the ability to capture biological variability, particularly for rare subtypes like malignant lymphomas. The cohort included 46 seminomas, 14 non-seminomas, and 5 malignant lymphomas, limiting statistical power and increasing susceptibility to bias in comparing ADC ratios. In addition, although the study included consecutive patients with MRI findings, selection bias could not be ruled out. Second, the methodology for selecting the region of interest introduced potential variability. Although ROI placement targeted the largest tumor cross-section and the contralateral testis for reproducibility, the single-observer design lacked multi-reader validation or standardized guidelines, limiting generalizability. Third, technical inconsistencies in MRI protocols were evident, as the study did not include phantom calibrations or inter-scanner corrections, raising concerns about the reproducibility of ADC measurements across different MRI systems. Additionally, as a retrospective single-center analysis, the findings may reflect institutional biases in imaging protocols or patient demographics. The absence of external validation cohorts further restricted broader applicability. Lastly, challenges in clinical implementation remain unresolved, including insufficient statistical robustness for rare subtypes, variability in ROI selection practices, and undefined thresholds for clinical decision-making. Future multicenter studies with larger cohorts, standardized imaging protocols, automated segmentation techniques of testes, and prospective validation are necessary to strengthen these preliminary findings.

## Conclusions

The present results demonstrate the efficacy of MRI with ADC values and histograms in the differentiation of testicular tumor subtypes. A pre-operative diagnosis of testicular tumor subtypes may result in more effective management of testicular tumors, including pre-operative counseling and early treatment planning.

## Data Availability

The data that support the findings of this study are not openly available due to reasons of sensitivity and are available from the corresponding author upon reasonable request.

## Abbreviations

ADC: Apparent diffusion coefficient
DWI: Diffusion-weighted imaging
MRI: Magnetic resonance imaging
ROI: Region of interest
T1WI: T1-weighted imaging
T2WI: T2-weighted imaging
